# Inclusive fitness benefits mitigate costs of cuckoldry to socially paired males

**DOI:** 10.1186/s12915-018-0620-6

**Published:** 2019-01-31

**Authors:** Aneesh P. H. Bose, Jonathan M. Henshaw, Holger Zimmermann, Karoline Fritzsche, Kristina M. Sefc

**Affiliations:** Institute of Biology, University of Graz, Universitätsplatz 2, 8010 Graz, Austria

**Keywords:** Multiple paternity, Inbreeding, Relatedness, Extra-pair, Male–male competition, Cichlid

## Abstract

**Background:**

In socially monogamous species, reproduction is not always confined to paired males and females. Extra-pair males commonly also reproduce with paired females, which is traditionally thought to be costly to the females’ social partners. However, we suggest that when the relatedness between reproducing individuals is considered, cuckolded males can suffer lower fitness losses than otherwise expected, especially when the rate of cuckoldry is high. We combine theoretical modeling with a detailed genetic study on a socially monogamous wild fish, *Variabilichromis moorii*, which displays biparental care despite exceptionally high rates of extra-pair paternity.

**Results:**

We measured the relatedness between all parties involved in *V. moorii* spawning events (i.e. between males and females in social pairs, females and their extra-pair partners, and paired males and their cuckolders), and we reveal that males are on average more related to their cuckolders than expected by chance. Queller–Goodnight estimates of relatedness between males and their cuckolders are on average *r* = 0.038 but can range up to *r* = 0.64. This also increases the relatedness between males and the extra-pair offspring under their care. These intriguing results are consistent with the predictions of our mathematical model, which shows that elevated relatedness between paired males and their cuckolders can be adaptive for both parties when competition for fertilizations is strong.

**Conclusions:**

Our results show how cuckoldry by relatives can offset males’ direct fitness losses with inclusive fitness gains, which can be substantial in systems where males face almost certain paternity losses.

**Electronic supplementary material:**

The online version of this article (10.1186/s12915-018-0620-6) contains supplementary material, which is available to authorized users.

## Background

Socially monogamous systems are characterized by the formation of pair bonds between males and females, though these pairs are not necessarily exclusive with respect to mating [1]. In particular, extra-pair males commonly sire some of paired females’ offspring. Relatedness among social pairs and extra-pair males influences the fitness outcomes of cuckoldry for all involved parties, according to inclusive fitness theory [2–5]. Despite this, few studies have comprehensively measured relatedness between all three pairwise relationships in socially monogamous systems, that is, between paired social partners, between females and their extra-pair partners, *and* between males and their cuckolders (see ref. [6]).

Empirical research on inbreeding in socially monogamous mating systems has commonly focused on inbreeding avoidance in birds. Females in many bird species solicit extra-pair copulations, which is often thought to result from a strategy to avoid inbreeding when they are paired with a highly related social partner [7–10] (but see ref. [11]). Such avoidance of inbreeding is often attributed to the costs of mating with relatives (e.g. inbreeding depression). Yet there is also a perplexing discrepancy between empirical observations in nature, which show that relatives are very rarely preferred as mates, and the theoretical literature, which expects inbreeding to be tolerated under a wide range of conditions owing largely to inclusive fitness benefits that can be gained by assisting relatives to propagate shared alleles [4, 5, 12].

In socially monogamous systems with extra-pair paternity, numerous pathways can lead to relatives being tolerated in reproductive contexts. First, selection may favour elevated relatedness between social partners. For example, sexual conflict often occurs between parents in biparental systems [13], though elevated within-pair relatedness may alleviate some of this conflict leading both parents to provide more care [14, 15] and thereby increase offspring fitness. Second, selection may favour elevated relatedness between females and their extra-pair mates. This is thought to occur when inclusive fitness benefits outweigh the costs of inbreeding depression and lost opportunities (if any) for the extra-pair males to find additional mates [5]. Third, while it is generally expected that males should avoid cuckolding relatives when given an option, alternate mating opportunities may not always be possible (e.g. in viscous populations). Alternatively, if a paired male faces inevitable paternity loss, such as when he is sperm depleted or unable to fully guard his mate, he may benefit by biasing extra-pair paternity towards related cuckolders. Further pathways can also lead to inbreeding tolerance irrespective of whether cuckoldry actually occurs. For example, elevated relatedness between social partners may be favoured if such pairings alleviate outbreeding costs [16, 17], or if males generally have limited mating opportunities [5]. Such complexity suggests that the substantial variation in reproductive behaviours and life histories among socially monogamous taxa may select for contrasting patterns of relatedness between social partners, genetic partners, and even sperm competitors. Thus, studying a diverse range of socially monogamous taxa, particularly those representing phylogenetically independent origins of the mating system, can provide a broader understanding of how inbreeding and relatedness shape reproductive behaviours.

Socially monogamous fish [1, 18] serve as an interesting comparison to more traditionally studied taxa such as birds because they differ dramatically in reproductive behaviours, parental care, and life history traits. Notably, females of many bird species exert a high degree of control over brood paternity [19] and can readily express preferences for extra-pair mates based on their degree of kinship (e.g. ref. [20–22]). In socially monogamous fishes, however, females are relatively less able to control which males gain paternity shares in their broods (but see ref. [23]; also see ref. [24, 25] for non-socially monogamous fishes). Furthermore, cuckoldry takes place at the nest site in many externally fertilizing fishes, whereas it occurs outside the territory boundaries in most birds. This can present paired male fish with the opportunity to selectively repel potential competitors.

In this study, we used a species of socially monogamous cichlid fish, *Variabilichromis moorii*, to investigate patterns of relatedness between paired males, paired females, and cuckolder males. Male–female social pairs defend rocky territories that they use for foraging and brood care [26–29]. Strong differentiation among populations of *V. moorii* in both mitochondrial and nuclear genomes on small geographic scales suggests philopatry of both sexes [30]. Thus, relatives may regularly interact with one another, affording kinship the opportunity to influence social bonds and mating decisions. Average paternity of the paired male is very low (e.g. 55.1%, ref. [31]). Up to 100% of nests can contain extra-pair offspring, and the number of extra-pair sires per brood can sometimes surpass 10 males [32]. We expected that the extreme rates of paternity loss in the *V. moorii* mating system would lead to patterns of elevated relatedness between paired males, females, and cuckolders. First, we predicted that paired males would exhibit elevated relatedness to their social mates as this could, in-part, counteract any sexual conflict between the parents [14, 15] that emerges after paternity losses. Second, we predicted that females would exhibit elevated relatedness to their extra-pair mates. Biasing paternity towards related cuckolders can generate inclusive fitness benefits for females when inbreeding depression is not too high [5]. Third, we predicted that males would exhibit elevated relatedness to their cuckolders. Paired males suffer large paternity losses in this system, which may be ameliorated if cuckoldry can be biased towards relatives. Furthermore, extra-pair males may be incentivized to cuckold their relatives if they experience reduced aggression while doing so and are thereby able to sire more offspring. We explore these arguments in more detail via a mathematical model, which was developed a posteriori in light of our empirical results.

To pursue these ideas, we used microsatellite genotyping of paired adults and their broods in the field. We calculated relatedness between paired males and females, between females and their extra-pair mates, and between males and their cuckolders, and we tested whether relatedness in any one of these pairings is greater than would be expected under random mating. Because of high densities of *V. moorii* at our study site, it was impossible to identify all cuckolders in the field, and so we reconstructed the genotypes of cuckolders based on the offspring that they sired in the broods of care-giving, paired parents. We also tested for deviations from expected parent–offspring relatedness due to relatedness within social pairs (e.g. a father’s expected relatedness to his within-pair young is 0.5 if he is unrelated to the mother but can be higher if they are related). Next, we measured the relatedness between females and their extra-pair offspring, which should be greater than 0.5 if females are related to their extra-pair mates. We also measured the relatedness between paired males and their mate’s extra-pair offspring, which should be positive if males are related to their cuckolders (while accounting for the measured relatedness between males and their social partners). Finally, we examined the spatial distribution of individuals in the wild to determine whether any patterns of relatedness that we uncovered could be due to active mechanisms of recognition or passive consequences of living in close proximity to kin.

## Results

### Paired males are more related to their cuckolders than expected by chance

Out of the 70 broods from which we sampled offspring, 60 contained fry unrelated to the brood-tending, paired male. Of these, 38 broods possessed sufficient extra-pair offspring to reconstruct a total of 74 cuckolder genotypes (each genotype consisting of at least 10 loci). When paired males experienced paternity losses, they were cuckolded by an average (± SD) of 1.95 ± 1.1 cuckolders (range = 1–5). Note that this average excludes broods with 0% paternity, because of the ambiguity in whether these were situations of 100% cuckoldry or territory takeovers (see the ‘Methods’ section; this occurred in 6 out of 70 broods).

**Fig. 1:**
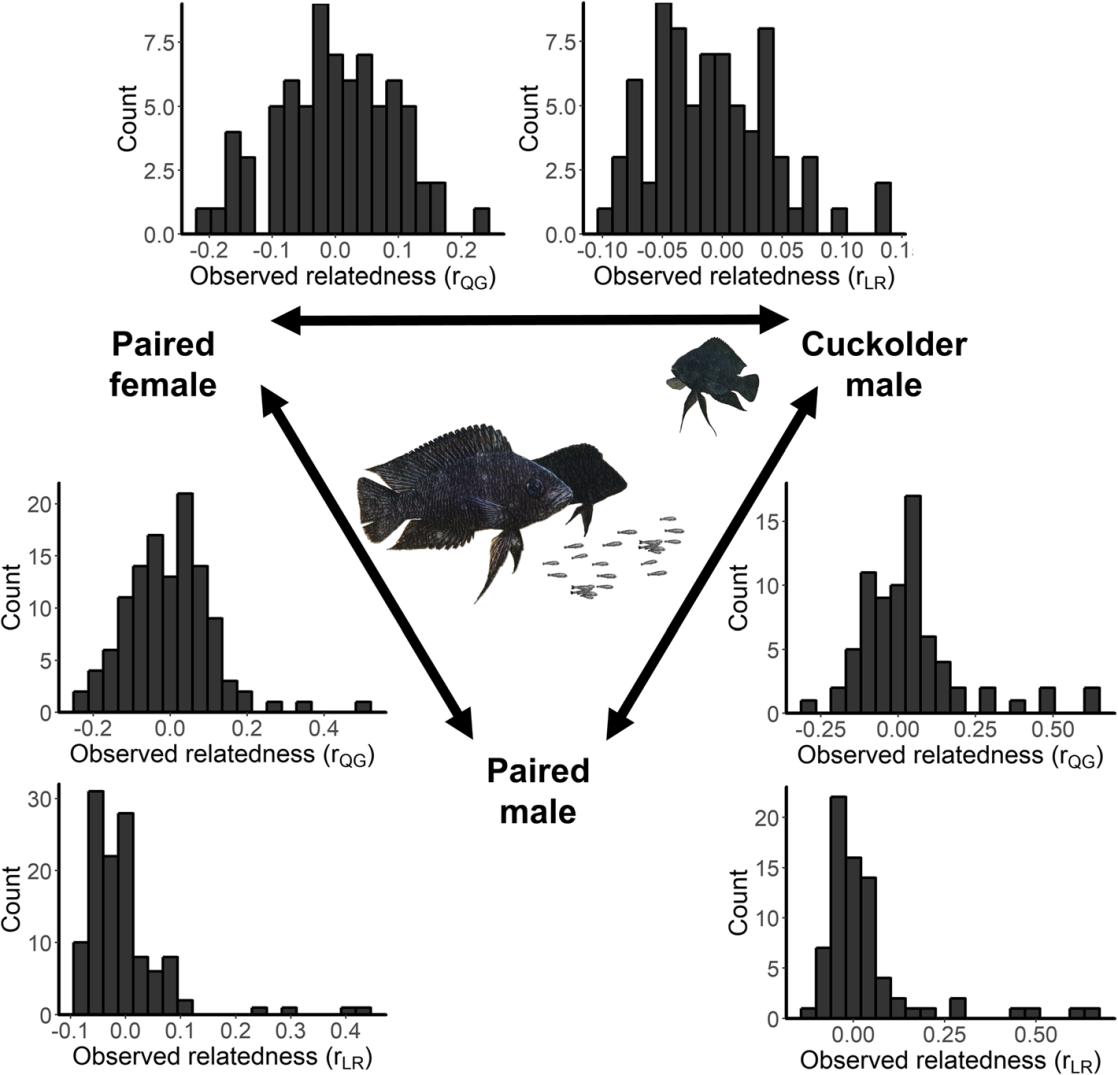
Bottom left histograms show the relatedness estimates between paired individuals. Top center histograms show mean relatedness estimates between paired females and their *set* of extra-pair (cuckolder) males. Bottom right histograms show mean relatedness between paired males and their *set* of cuckolders. Relatedness estimates were calculated following Queller and Goodnight [71], *r*_QG_, and Lynch and Ritland [72], *r*_LR_

**Fig. 2:**
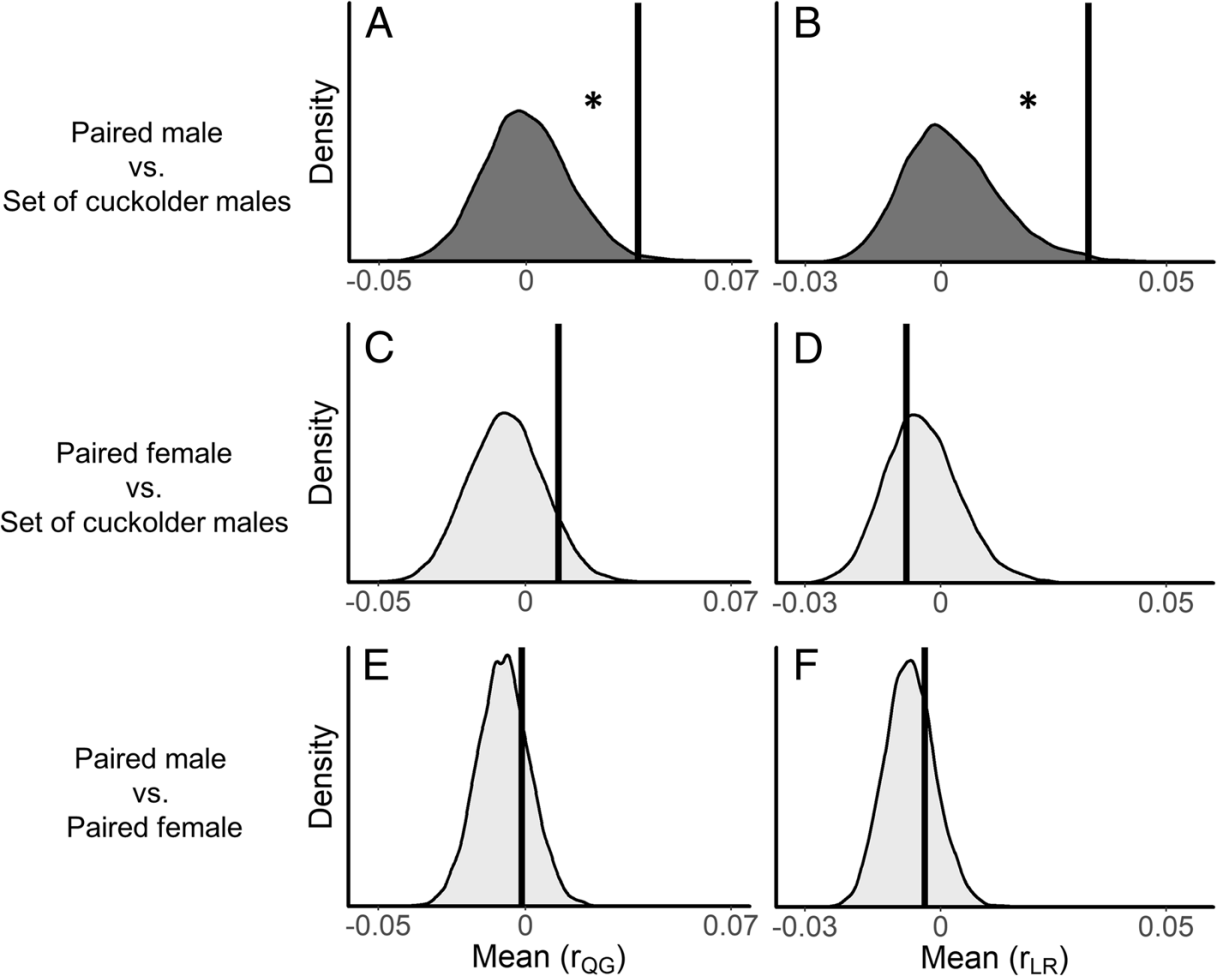
Density plots showing null distributions for mean pairwise relatedness estimates derived from the randomization tests described in the methods. **a** and **b** panels respectively show null distributions of *r*_QG_ and *r*_LR_ for paired males versus their cuckolders. **c** and **d** panels show null distributions of *r*_QG_ and *r*_LR_ for paired females versus their extra-pair males (i.e. cuckolders). **e** and **f** panels show null distributions of *r*_QG_ and *r*_LR_ for paired males versus their female partners. Vertical black bars indicate our observed values. Darker shading with asterisk indicates significant results after implementing the Benjamini–Hochberg procedure for controlling false discovery rates, here set to 10%

Figure 1 illustrates the distributions of relatedness estimates that we observed in the field between paired males, females, and cuckolders, while Table 1 provides summary statistics for our observed and permuted data as well as the computed *p* values. Paired males were on average *more* related to their set of cuckolders than expected under random mating (*N* = 38 paired males, *N* = 73 cuckolders, Fig. 2a, b). Paired females, on the other hand, were not more related to their set of extra-pair males (cuckolders) than expected by chance (*N* = 39 paired females, *N* = 74 cuckolders, Fig. 2c, d). Average relatedness between paired males and their females also did not differ from the expectations of random mating (*N* = 119 social pairs, Fig. 2e, f). Our observed variance and skewness values did not differ from expectations of random mating for any of the pairings that we investigated (Table 1, Additional file 1: Figure S1).

**Table 1:** Mean, variance, and skewness of the observed distributions of pairwise relatedness estimates between all three parties in the socially monogamous mating system of *V. moorii*

	Mean	Variance	Skewness
Paired male vs. set of cuckolder males	*r*_QG_, 0.038 (0.00030) *p* = 0.0088*	*r*_QG_, 0.012 (0.0093) *p* = 0.15	*r*_QG_, 0.89 (0.63) *p* = 0.26
	*r*_LR_, 0.033 (0.0025) *p* = 0.0093*	*r*_LR_, 0.0067 (0.0048) *p* = 0.17	*r*_LR_, 0.95 (1.60) *p* = 0.65
Paired female vs. Set of cuckolder males	*r*_QG_, 0.011 (− 0.0066) *p* = 0.085	*r*_QG_, 0.0061 (0.0070) *p* = 0.67	*r*_QG_, 0.21 (0.16) *p* = 0.41
	*r*_LR_, − 0.0076 (− 0.0040) *p* = 0.65	*r*_LR_, 0.0015 (0.0028) *p* = 0.91	*r*_LR_, 0.46 (1.10) *p* = 0.81
Paired male vs. Paired female	*r*_QG_, − 0.0012 (− 0.0074) *p* = 0.24	*r*_QG_, 0.012 (0.011) *p* = 0.16	*r*_QG_, 1.00 (0.29) *p* = 0.072
	*r*_LR_, − 0.0035 (− 0.0067) *p* = 0.28	*r*_LR_, 0.0065 (0.0038) *p* = 0.057	*r*_LR_, 3.09 (1.44) *p* = 0.090

Averages for the mean, variance, and skewness of the permuted null distributions are given in parentheses, while *p* values are given beneath. *p* values marked with an asterisk indicate statistically significant results after applying the Benjamini–Hochberg procedure for controlling false discovery rates, here set to 10%

### Cuckoldry between relatives increases the relatedness between paired males and extra-pair fry

Whereas the above analyses were restricted to cuckolders whose genotypes could be well-reconstructed (see the ‘Methods’ section), the following analyses of parent–offspring relatedness allowed us to consider all captured offspring. Consistent with our finding of elevated relatedness between paired males and their cuckolders, the relatedness estimates between paired males and the extra-pair fry in their broods were significantly greater than 0 after accounting for any deviation from random allele sharing between the paired male and female (LMM: intercept-term, r_QG_, est. ± se = 0.024 ± 0.0079, *t*_743_ = 3.02, *p* = 0.0026; r_LR_, est. ± se = 0.023 ± 0.0080, *t*_743_ = 2.93, *p* = 0.0035). Neither the relatedness estimates between paired females and their extra-pair fry (intercept-term, r_QG_, est. ± se = 0.0041 ± 0.0054, *t*_578_ = 0.74, *p* = 0.46; r_LR_, est. ± se = − 0.011 ± 0.011, *t*_578_ = − 0.99, *p* = 0.32) nor the relatedness estimates between paired males and their within-pair fry (intercept-term, r_QG_, est. ± se = 0.0032 ± 0.0069, *t*_1018_ = 0.46, *p* = 0.65; r_LR_, est. ± se = − 0.0053 ± 0.0086, *t*_1018_ = − 0.62, *p* = 0.53) differed significantly from 0.5.

### Potential cuckolder males remain near to related paired males in the field

**Fig. 3:**
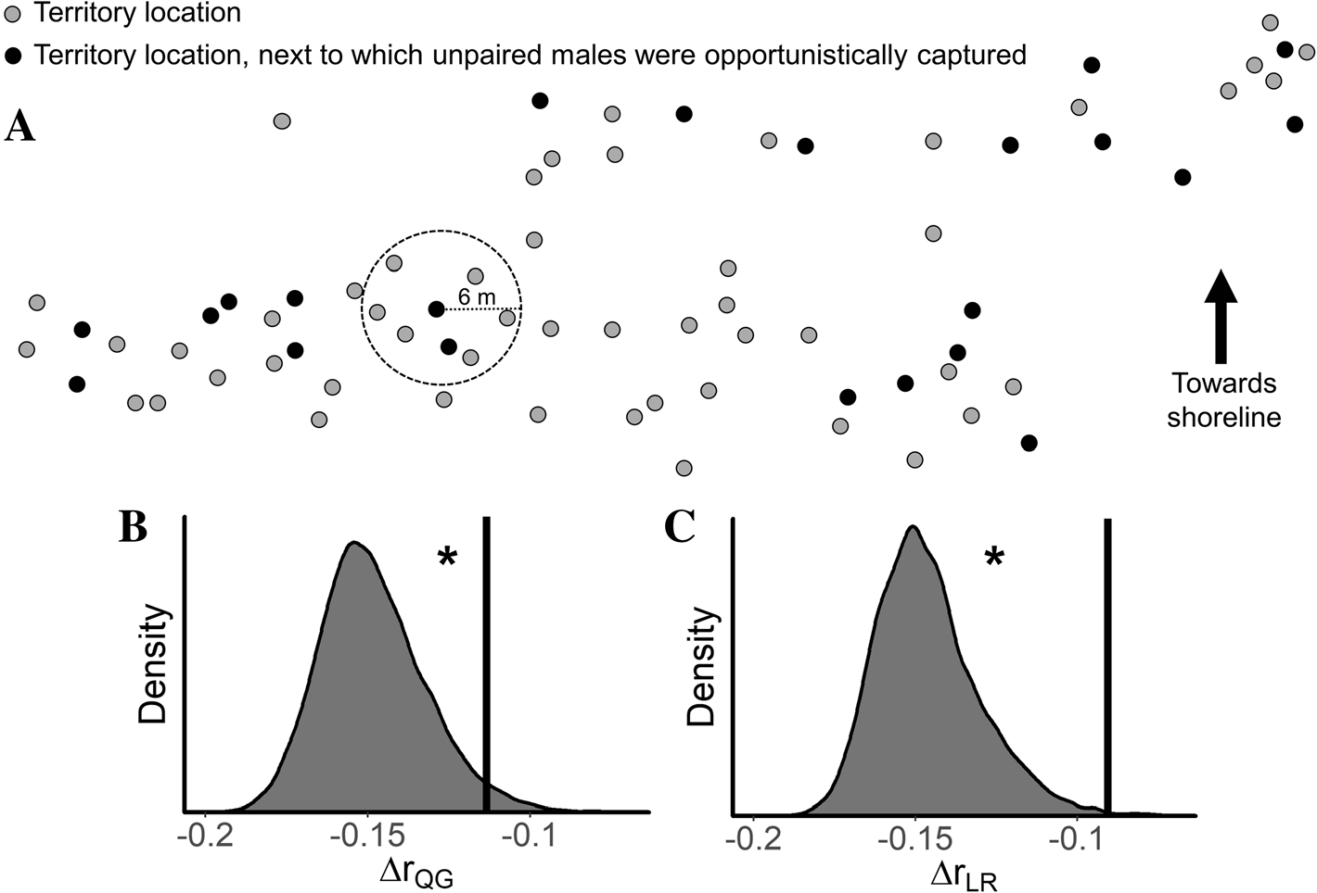
**a** Map showing spatial distribution of *V. moorii* breeding territories in our study quadrat as sampled in the dry season (October 2015). Both grey and black circles represent the locations of breeding pairs’ territories. While the grey circles represent the territories where the breeding pair was caught, the black circles represent the territories next to which unpaired males (i.e. potential cuckolders) were also caught. The dotted circle provides a measure of scale (in this case, 6 m) and can be used to count how many breeding territories are within a *X* m proximity of the unpaired male. **b** and **c** panels show the permuted null distributions for Δ*r* (see the ‘Methods’ for details) for each relatedness estimator separately and calculated at a radius of 6 m. The vertical bars indicate our observed Δ*r* values. Asterisk indicates significant results after implementing the Benjamini–Hochberg procedure for controlling false discovery rates, here set to 10%. Our observed Δ*r* values were higher than expected by chance for all radii tested from 6 to 10 m

Bose et al. [31] recently showed that nearly all cuckoldry in the *V. moorii* system is perpetrated by unpaired males. Here, we found that unpaired males were not captured in the *immediate* vicinity of any paired males to whom they shared high relatedness estimates. That is, the observed Δ*r*_QG_ and Δ*r*_LR_ values were not higher than expected by chance for any radius that we tested up to 5 m from each unpaired male (all *p* > 0.068, see the ‘Methods’ section, Additional file 1: Table S1). However, we found that unpaired males maintained a more moderate distance to some paired males with comparatively high relatedness estimates (Fig. 3). Figure 3a illustrates the spatial distribution of *V. moorii* territories in the study quadrat including the territories next to which unpaired males were captured. Our observed Δ*r*_QG_ and Δ*r*_LR_values were nearly always higher than expected by chance for radii 6 m (Fig. 3b and c) and higher (all *p* < 0.05 for each radius from 6 to 10 m, excepting Δ*r*_QG_ at 7 m where *p* = 0.063; Additional file 1: Table S1). Lastly, we did not detect any significant correlation between spatial separation and relatedness estimates between paired, territory-holding males in our quadrat (*r*_QG_, Mantel *r* = 0.020, *p* = 0.21; *r*_LR_, Mantel *r* = 0.0034, *p* = 0.43; see Additional file 1: Figure S2).

## Mathematical model

Given these intriguing empirical results, we built a game-theoretic model to further illuminate the evolutionary interests of both paired and cuckolder males. We consider the conditions under which it is adaptive for (1) paired males to tolerate related cuckolders and (2) cuckolders to target related nest-holders (i.e. paired males). We assume a large population with sufficient mixing such that most competitive interactions occur between unrelated individuals. Potential cuckolders are drawn from two sources: a large pool of males that are unrelated to the paired male and a small group of closely related males. Our model assumes that cuckolders can identify (at least some) related paired males and vice versa. Aside from relatedness, all paired males are assumed identical, as are all cuckolders.

Paired males have two possible strategies: either defend their mate’s eggs against all cuckolders indiscriminately, or defend preferentially against unrelated males. Similarly, cuckolders can choose to preferentially target either related or unrelated paired males. We assume that genes determining the behaviour of paired males and cuckolders are not expressed by males playing the opposite role, which simplifies the analysis of inclusive fitness [33]. Paired males who defend discriminately are assumed to have reduced defensive capability, due e.g. to the cognitive burdens of kin recognition or strategic complications of defending against some males more than others. This cost is paid regardless of whether a related cuckolder is actually present among the males attempting to obtain fertilizations during a spawning event.

Three points help to clarify the evolutionary interests of both types of male. First, if paired males are capable of driving off all cuckolders, then it is always adaptive to do so. We consequently assume that paired males only tolerate related cuckolders when there are more potential cuckolders than can be driven away. Second, cuckolders should always attempt to cuckold related males if no other target is available, assuming the costs of cuckoldry (e.g. risk of injury) are low. We thus focus on a cuckolder’s choice between a related and an unrelated male target. Third, if paired males defend indiscriminately against all incomers, then there is no direct fitness benefit to cuckolders of targeting a relative instead of a non-relative. There is, however, an indirect fitness cost due to paternity loss of the paired male relative. We therefore restrict our analysis of cuckolders’ choices to populations where paired males discriminate in favour of related cuckolders.

### Model structure

We focus here on the case where only one potential cuckolder is related to the paired male (but see Additional file 1: Supplementary materials for a partial relaxation of this assumption). We write *r* for the relatedness coefficient between the paired male and the related cuckolder. The average relatedness between paired males and ‘unrelated’ cuckolders is assumed to be zero, consistent with the assumption of a large and well-mixed population.

For any given spawning event, the number of unrelated potential cuckolders *C* is assumed to follow a Poisson distribution with mean *μ*_*C*_. A related male attempts to cuckold with probability *f*. Since there is at most one related cuckolder, the total number of potential cuckolders is either *C* or *C* + 1. The number of potential cuckolders that the paired male can drive off is also a random variable *D*, which follows a Poisson distribution with mean *μ*_D_ (for males that defend indiscriminately) or (1 − *a*)*μ*_D_ (for males that defend preferentially against non-relatives). The parameter 0 ≤ *a* < 1 represents the cost of discriminate defense.

The nest-holder drives off all potential cuckolders when *D* ≥ *C* (if no related male is present) or *D* ≥ *C* + 1 (if a related male is present). In this case, he fertilizes all of his mate’s eggs himself. On the other hand, if *D* < *C* or *D* < *C* + 1 respectively, then paternity is shared between the paired male and the successful cuckolders, with all successful males having an equal chance of fertilizing any given egg [34]. Consequently, the expected proportion of eggs fertilized by the paired male is $\frac{1}{1+C-D}$ when no related male is present or $\frac{1}{2+C-D}$ when a related male is present. We derive the average relatedness of the paired male to all of his successful cuckolders (both related and unrelated) in Additional file 1: Supplementary materials.

### Fitness of the paired male

We now calculate the inclusive fitness consequences for a paired male of defending indiscriminately versus preferentially tolerating a related cuckolder. Tolerance is adaptive if it increases the sum of (1) the paired male’s direct fitness gain (i.e. the number of offspring sired in the relevant fertilization event) and (2) the cuckolder’s direct fitness gain, weighted by the relatedness coefficient *r*. This is a phenotypic form of Hamilton’s rule [35, 36] (i.e. an action *X* directed towards a relative is selected for over an action *Y* if *rB*_*X*_ − *C*_*X*_ > *rB*_*Y*_ − *C*_*Y*_, where the *B*_*i*_ represent the benefits to the recipient of each action, *C*_*i*_ are the costs to the actor, and *r* is the coefficient of relatedness between the actor and recipient). Calculating the inclusive fitness consequences of a decision, as we do, avoids the thornier accounting issues that arise when defining inclusive fitness as a property of an individual (see ref. [37]).

Since the number of unrelated cuckolders *C* and the number of cuckolders that can be driven off *D* both follow Poisson distributions, their difference *C* − *D* follows a Skellam distribution with probability mass function [38]:

1 $$ {s}_k\left({\mu}_C,{\mu}_D\right)=\mathrm{\mathbb{P}}\left(C-D=k\right)={e}^{-\left({\mu}_C+{\mu}_D\right)}{\left(\frac{\mu_C}{\mu_D}\right)}^{k/2}{I}_k\left(2\sqrt{\mu_C{\mu}_D}\right), $$

where ${I}_k(x)=\frac{1}{\pi }{\int}_0^{\pi }{e}^{x\cos \theta}\cos \left( k\theta \right)\  d\theta$ is the modified Bessel function of the first kind. Supposing *C* − *D* = *k*, we have two scenarios. First, if no related male is present (with probability 1 − *f*), then the paired male expects to fertilize a proportion $\frac{1}{1+{k}^{+}}$ of his mate’s eggs, where we write *x*^+^ = max {*x*, 0}. Second, if a related male is present (with probability *f*), then the paired male’s expected fertilization success is given by $\frac{1}{1+{\left(k+1\right)}^{+}}$. The direct fitness gain of a paired male that defends indiscriminately is then:

2 $$ {W}_{\mathrm{indisc}}^{\mathrm{d}}={\sum}_{k=-\infty}^{\infty }{s}_k\left({\mu}_C,{\mu}_D\right)\left[\left(1-f\right)\left(\frac{1}{1+{k}^{+}}\right)+f\left(\frac{1}{1+{\left(k+1\right)}^{+}}\right)\right] $$

For a male that discriminates in favour of related cuckolders, the direct fitness gain is given by the same expression except that his defense capability is reduced by a factor of (1 − *a*):

3 $$ {W}_{\mathrm{d}\mathrm{isc}}^{\mathrm{d}}=\sum \limits_{k=-\infty}^{\infty }{s}_k\left({\mu}_C,\left(1-a\right){\mu}_D\right)\left[\left(1-f\right)\left(\frac{1}{1+{k}^{+}}\right)+f\left(\frac{1}{1+{\left(k+1\right)}^{+}}\right)\right] $$

We now consider the indirect fitness obtained by paired males when a related cuckolder fertilizes some of their mate’s eggs. We write *p*_*c*_(*μ*_*C*_) for the probability that there are exactly *C* = *c* unrelated cuckolders and *p*_*d*_(*μ*_*D*_) for the probability that the paired male can drive off at most *D* = *d* cuckolders, where ${p}_k\left(\lambda \right)=\frac{\lambda^k{e}^{-\lambda }}{k!}$ is the probability mass function for the Poisson distribution. If a related cuckolder is present (with probability *f*), then there are *c* + 1 cuckolders in total, of which *c* + 1 − *d* are successful in gaining paternity. When the paired male defends indiscriminately, the probability that the related cuckolder is successful is thus $\frac{c+1-d}{c+1}$. The related cuckolder’s expected share of paternity in this case is $\frac{1}{c+2-d}$. Hence, the paired male’s indirect fitness gain due to fertilizations by related cuckolders is:

4 $$ {W}_{\mathrm{i}\mathrm{ndisc}}^{\mathrm{i}}=f\cdotp r{\sum}_{c=0}^{\infty }{p}_c\left({\mu}_C\right){\sum}_{d=0}^c{p}_d\left({\mu}_D\right)\left(\frac{c+1-d}{c+1}\right)\left(\frac{1}{c+2-d}\right) $$

For paired males that defend discriminately, we assume that whenever a related male is present and some subset of cuckolders is successful in obtaining paternity, then the related male is always among that subset. The indirect fitness gain of a discriminating paired male is then:

5 $$ {W}_{\mathrm{disc}}^{\mathrm{i}}=f\cdotp r{\sum}_{c=0}^{\infty }{p}_c\left({\mu}_C\right){\sum}_{d=0}^c{p}_d\left(\left(1-a\right){\mu}_D\right)\left(\frac{1}{c+2-d}\right) $$

It is clear that ${W}_{\mathrm{indisc}}^{\mathrm{d}}>{W}_{\mathrm{d}\mathrm{isc}}^{\mathrm{d}}$ but ${W}_{\mathrm{i}\mathrm{ndisc}}^{\mathrm{i}}<{W}_{\mathrm{disc}}^{\mathrm{i}}$. In other words, defending discriminately reduces a paired male’s direct fitness but increases his indirect fitness. Discriminating is adaptive when ${W}_{\mathrm{d}\mathrm{isc}}^{\mathrm{d}}+{W}_{\mathrm{d}\mathrm{isc}}^{\mathrm{i}}>{W}_{\mathrm{i}\mathrm{ndisc}}^{\mathrm{d}}+{W}_{\mathrm{i}\mathrm{ndisc}}^{\mathrm{i}}$.

### Fitness of the cuckolder

We now consider the inclusive fitness consequences for unpaired males of targeting either a related male or an unrelated male. We assume a population where all paired males discriminate in favour of related cuckolders. By similar logic to above, the direct fitness gain of a cuckolder targeting a related male is:

6 $$ {W}_{\mathrm{rel}}^{\mathrm{d}}={\sum}_{c=0}^{\infty }{p}_c\left({\mu}_C\right){\sum}_{d=0}^c{p}_d\left(\left(1-a\right){\mu}_D\right)\left(\frac{1}{c+2-d}\right) $$

For a cuckolder targeting an unrelated male, the direct fitness gain is:

7 $$ {W}_{\mathrm{unrel}}^{\mathrm{d}}={\sum}_{c=0}^{\infty }{p}_c\left({\mu}_C\right){\sum}_{d=0}^c{p}_d\left(\left(1-a\right){\mu}_D\right)\left(\frac{c+1-d}{c+1}\right)\left(\frac{1}{c+2-d}\right) $$

If the cuckolder targets his relative, his indirect fitness gain via the paired male’s fertilization success is:

8 $$ {W}_{\mathrm{rel}}^{\mathrm{i}}=r{\sum}_{k=-\infty}^{\infty }{s}_k\left({\mu}_C,\left(1-a\right){\mu}_D\right)\frac{1}{1+{\left(1+k\right)}^{+}} $$

If the cuckolder instead targets an unrelated male, his indirect fitness gain is:

9 $$ {W}_{\mathrm{unrel}}^{\mathrm{i}}=r{\sum}_{k=-\infty}^{\infty }{s}_k\left({\mu}_C,\left(1-a\right){\mu}_D\right)\frac{1}{1+{k}^{+}} $$

It is clear that ${W}_{\mathrm{rel}}^{\mathrm{d}}>{W}_{\mathrm{unrel}}^{\mathrm{d}}$ but ${W}_{\mathrm{rel}}^{\mathrm{i}}<{W}_{\mathrm{unrel}}^{\mathrm{i}}$. Targeting a relative increases a cuckolder’s direct fitness, because the cuckolder benefits from the paired male’s tolerance. On the other hand, he also obtains paternity at the expense of his relative, which reduces his indirect fitness. Targeting a related paired male is adaptive when ${W}_{\mathrm{rel}}^{\mathrm{d}}+{W}_{\mathrm{rel}}^{\mathrm{i}}>{W}_{\mathrm{unrel}}^{\mathrm{d}}+{W}_{\mathrm{unrel}}^{\mathrm{i}}$.

### Model results

**Fig. 4:**
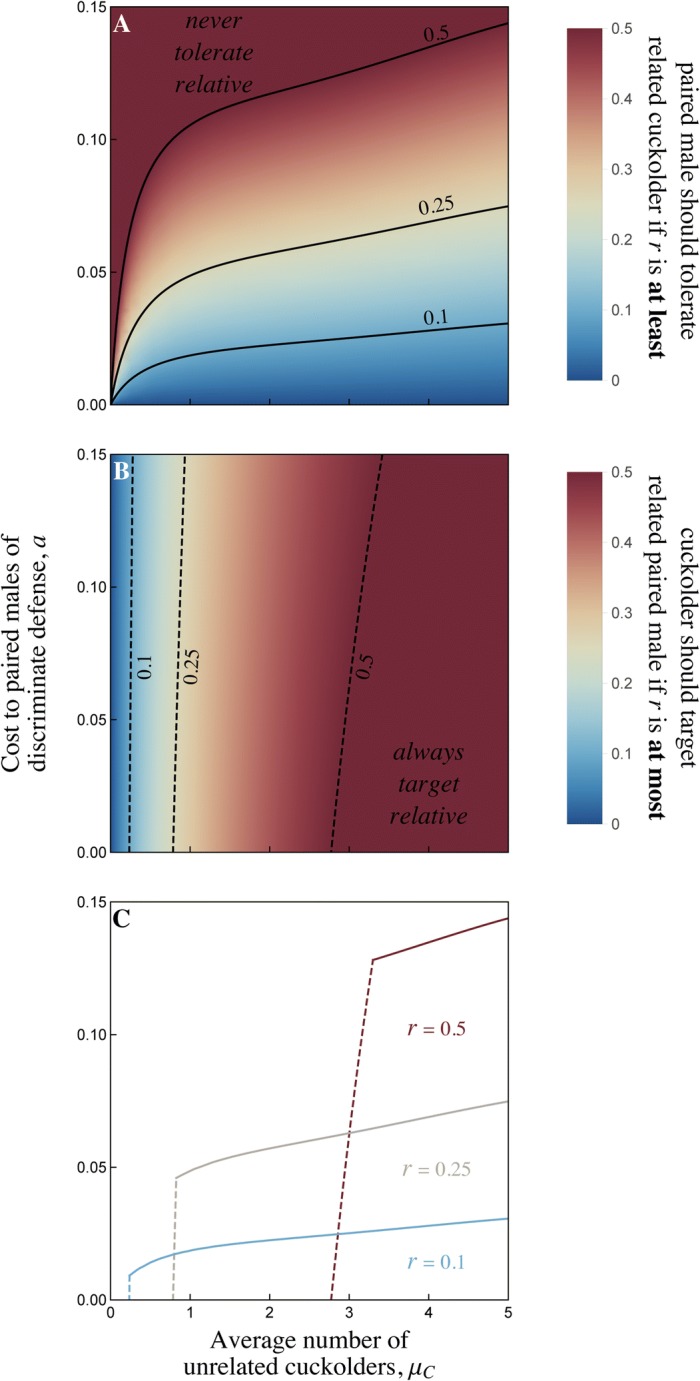
When should paired males tolerate related cuckolders and when should cuckolders target related paired males? **a** Tolerance of related cuckolders by paired males is adaptive when the cost of discriminate defense is not too high (small *a*); when on average many unrelated cuckolders target a spawning event (high *μ*_*C*_); and when the relatedness coefficient between the paired male and the related cuckolder is *sufficiently* high (large *r*). For example, if the cuckolder is a half-brother, tolerance is adaptive in the region *below* the contour marked 0.25. **b** Cuckolders should target related paired males when the cost to the paired male of discriminate defense is not too high (small *a*); when on average many unrelated cuckolders target a spawning event (high *μ*_*C*_); and when the relatedness coefficient between the cuckolder and the paired male is *not too* high (small *r*). For example, if the paired male is a half-brother, the cuckolder should target him rather than a non-relative in the region *to the right* of the contour marked 0.25. Note that this panel assumes that the paired male tolerates the related cuckolder whenever he is unable to drive off all potential cuckolders. **c** Parameter regions where paired males should tolerate relatives (below solid lines) *and* cuckolders should target relatives (to the right of dashed lines), combining the results of panels **a** and **b**. The relatedness between paired male and cuckolder is *r* = 0.5 (maroon lines); *r* = 0.25 (beige lines); and *r* = 0.1 (blue lines). All panels are shown with a probability *f* = 0.5 that the related cuckolder is present at a given spawning event, and a mean number *μ*_*D*_ = 1 of cuckolders that the paired male can drive off. Note that the number of successful cuckolders may be much smaller than the number of potential cuckolders

If many unrelated males attempt to cuckold an average spawning event (i.e. if *μ*_*C*_ is large), then the paired male and his cuckolder relative compete for fertilizations not only with each other, but also with all the unrelated cuckolders. This favours tolerance by the paired male, because paternity gains by his relative are more likely to come at the expense of an unrelated cuckolder, rather than the paired male himself (Fig. 4a). For the same reason, cuckolders should prefer to target related paired males (or at least the subset of related males that will tolerate them) when *μ*_*C*_ is large (Fig. 4b). Note that the number of successful cuckolders, as measured in our empirical study, may be much smaller than the number of potential cuckolders (see Additional file 1: Supplementary materials).

If the average number of unrelated cuckolders is small, then the interests of the paired male and cuckolder are almost directly opposed, regardless of their population-level relatedness (i.e. we have a nearly zero-sum game: ref. [39]). Paired males should consequently defend indiscriminately (Fig. 4a) and cuckolders should prefer to target unrelated paired males (Fig. 4b) when sperm competition is low. The role of sperm competition in determining the scope for cooperation among related males is analogous to the ‘scale of competition’ in inclusive fitness models, where altruism towards kin is predicted only when there is sufficient competition between unrelated individuals [40, 41]. Unsurprisingly, regardless of the level of sperm competition, paired males should only tolerate relatives if discriminate defense is not too costly (i.e. the reduction in defensive capability *a* is small: Fig. 4a).

The effect of relatedness between the paired males and cuckolders is subtle. By defending discriminately, paired males incur a direct fitness cost via the reduction in their defense capabilities. However, this is offset by the indirect fitness benefit of increased fertilization success by related cuckolders. Tolerance of relatives is favoured when the indirect fitness benefit is large, which occurs when relatedness is high (Fig. 4a). In contrast, cuckolders obtain a direct fitness *benefit* by targeting a related paired male if that paired male discriminates in the cuckolder’s favour. This is balanced by an indirect fitness *cost* of stealing paternity from a relative. Cuckolders should consequently only target a related paired male if the relatedness coefficient is *not too* high (Fig. 4b; again, this assumes that the cuckolder can count on the paired male’s tolerance). At the population level, elevated relatedness between paired males and cuckolders is expected to be observed when both behaviours (i.e. paired males’ tolerance and cuckolders’ targeting of relatives) are selected for. This occurs over a broad parameter region where the density of unrelated cuckolders is high and the cost of discriminate defense is low (Fig. 4c).

## Discussion

In this study, we describe the relatedness structure for *all three* pairwise relationships that exist in a typical socially monogamous mating system, namely between paired males and females, between females and their extra-pair mates, and also between males and their cuckolders. However, the *V. moorii* mating system is also atypical in that it is characterized by extremely high rates of cuckoldry. This motivated us to test for patterns of relatedness between individuals because of the potential for inclusive fitness benefits that mitigate the costs of cuckoldry. We show here that although paired *V. moorii* males lose substantial paternity to cuckolders in the wild, they are also on average more related to these cuckolders than would be expected by chance alone. Prior to ours, almost no study to date has shown, or indeed tested for, relatedness between males and their cuckolders in a non-cooperatively breeding species (see ref. [6]).

We found that the average relatedness between a paired male and his *set* of cuckolders (ranging from one to five cuckolders per male) was *r*_GQ_ = 0.038 and *r*_LR_ = 0.033 (Fig. 2a and b). These averages are significantly higher than chance because some cuckolders were apparently close kin with their cuckolds (i.e. high relatedness estimates), though most cuckolders were apparently unrelated (i.e. low relatedness estimates). In fact, the average relatedness between each paired male and his *most* related cuckolder was *r*_QG_ = 0.12 (range = − 0.17 to 0.65) and *r*_LR_ = 0.098 (range = − 0.08 to 0.67). We also found that paired males were appreciably related to the extra-pair offspring in their broods owing to the males’ elevated relatedness to their cuckolders. Although the average relatedness between males and their cuckolders appears low at first glance, the high proportion of extra-pair fertilizations that occur in this species means that the inclusive fitness benefits are not negligible. We calculated the fitness consequences for a *V. moorii* male that is cuckolded by relatives versus non-relatives by assuming an average paternity loss of 44.9% (value taken from ref. [31]). We calculated that cuckoldry by individuals of *r* = 0.038 (*r*_QG_, taken from the current study) should increase male fitness by 3.1% compared to an equivalent amount of cuckoldry by non-relatives (i.e. if *r* were zero; see Additional file 1: Supplementary materials). This means that the fitness value of a brood (i.e. its ‘allelic value’, sensu ref. [6]) cannot simply be assessed by counting the number of offspring that a male sires; the alleles shared between the male and his cuckolders must also be taken into account. This also means that if cuckoldry is inevitable, or at least highly likely, as it is in *V. moorii*, then males may benefit by allowing related cuckolders to join in a spawning event as this would enable them to maintain a higher average relatedness to their brood. In some species, the inclusive fitness benefits offered by extra-pair offspring may even be high enough to affect how selection acts on males to flexibly reduce paternal investment in response to cuckoldry [6]. It will be important to incorporate such variation into future theoretical models of inbreeding, parentage, and parental investment [42].

We found a spatial pattern in the relatedness structure between paired males and unpaired males, and this provides context for our findings that relatedness between cuckolds and cuckolders is elevated in this system. We focused here on unpaired males because they commit the vast majority of cuckoldry in this species [31]. We showed that the spatial distribution of unpaired males relative to paired males is not random with respect to relatedness coefficients; within a reasonable radius or ‘swimming distance’, an unpaired male will, more often than expected by chance, find a paired male with whom he shares a higher relatedness estimate than with many other paired males in the quadrat. Unpaired males may therefore actively choose to remain in proximity to certain paired males based on their degree of kinship. Our mathematical model predicts that if paired males exhibit less aggression towards related competitor males during a spawning event, then this could incentivize unpaired males to seek out related cuckolds. By reducing their aggression towards related cuckolders, paired males may also benefit from the inclusive fitness benefits of sharing paternity with a related cuckolder rather than a non-relative. Reduced aggression leading to increased fertilization success by related cuckolders could similarly explain our results even if unpaired males cuckolded indiscriminately within their home ranges. Future studies involving direct observations of the interactions between paired males and their potential cuckolders during spawning events will be valuable for elucidating the mechanisms that underlie cuckoldry among male relatives in *V. moorii*. Meanwhile, our analyses showed no other spatial patterns in the relatedness between territory-holding, paired males in our study quadrat. Paired males apparently live in close proximity to both relatives and non-relatives alike in the wild.

We did not find that *V. moorii* adults form social pairs based on relatedness. We had expected that individuals would prefer to pair with relatives (especially if inbreeding depression is low) in order to reduce sexual conflict over parental care and thereby potentially benefit offspring fitness [14, 15]. We also did not find that *V. moorii* females were related to their extra-pair mates. Theory generally predicts that females should seek extra-pair copulations with males that are *more* related to them than their social partners, specifically when the inclusive fitness benefits of doing so outweigh the costs of inbreeding [5]. Similarly, if cuckoldry is unavoidable and costly to females (e.g. when cuckolders are genetically inferior males), then biasing extra-pair paternity towards related cuckolders may also be a way for females to recoup some of the fitness losses associated with mating with these non-preferred males. Future studies may therefore want to quantify the severity of inbreeding depression in this system to investigate whether it can deter pairing and mating between relatives. Furthermore, even if females exhibit a preference for related extra-pair males, this could be masked when one considers the cuckolder’s perspective; when neither extra-pair reproduction nor inbreeding are particularly costly, extra-pair males should be unwilling to turn down cuckoldry opportunities regardless of their kinship with the females. Thus, the degree to which females can control the identity of their extra-pair mates (i.e. cuckolders) is also critical to consider. In general, females of most externally fertilizing fishes are assumed to have little control, but the potential for female choice or cryptic choice at the gametic level to influence patterns of paternity should still be given due attention [23–25]. Future studies in *V. moorii* may wish to investigate whether females can bias paternity towards particular male genotypes or if their pairing preferences are correlated with the genetic structure of paired males’ neighborhoods.

That indirect fitness gains can offset direct fitness losses is not a new idea (e.g. ref. [6]), and inclusive fitness is often invoked when attempting to explain cases of same-sex individuals behaving altruistically when they are otherwise expected to compete (reviewed in ref. [43]). Theoretical work has established that relatedness among reproductive rivals should diminish their competitiveness, particularly when populations are group-structured and competition causes harm to individuals of the opposite sex [44]. This concept is most commonly visualized as males competing over females and has garnered empirical support; for example, competition for females is lower within groups of related compared to unrelated males in fruit flies, *Drosophila melanogaster* [45], and seed beetles, *Callosobruchus maculatus* [46]. In certain avian species, kinship among females is also known to underlie some surprising acts of altruism, such as during conspecific brood parasitism in which females lays their eggs in the nests of female relatives who then care for the young (reviewed in ref. [47]). Intriguingly, the phenomenon of female conspecific brood parasitism draws many parallels to cuckoldry in paternal or biparental caregiving species, such as *V. moorii*, in which males provide care for offspring that they may not have sired. Valuable avenues of future research include (i) directly observing the behavioural interactions between spawning males and females in *V. moorii* to establish the mechanism giving rise to our observed patterns and (ii) broadly determining whether elevated male-cuckolder relatedness is taxonomically widespread, and if so, whether there are factors that consistently select for this pattern across species.

How kin-selection and relatedness influence reproductive competition has already been well-studied in the contexts of male reproductive cooperation [43]. Relatedness and the formation of kin-groups or neighborhoods is also thought to play a major role in the evolution of cooperative breeding in certain taxa [48, 49] (but see refs [50–52].). Interestingly, *V. moorii* is not a cooperative breeder but other species within its tribe are (tribe Lamprologini, e.g. *Neolamprologus pulcher*, *N. savoryi*, *Julidochromis ornatus, N. multifasciatus*, and *N. obscurus* [53]), and helpers are often related to at least one of the breeders [54, 55]. Cooperative breeding has evolved multiple times independently within this tribe [52, 56]. Shared ancestry and/or shared ecological features among Lamprologines may predispose these species towards tolerance of relatives in reproductive contexts, which may have further evolved into cooperative behaviours in some of them.

## Conclusions

By studying how relatedness between reproductive competitors conveys indirect fitness benefits, we merge sexual selection theory with inclusive fitness theory. It is rare for empirical studies on socially monogamous non-cooperative systems to describe the relatedness structure between males and their cuckolders, and rarer still for these studies to find that these males have elevated relatedness to one another. Here, we show that in a large wild population of free-living and spatially unrestrained individuals, paired males are on average more related to their cuckolders than expected by chance, and this leads them to be more related to the extra-pair offspring in their broods than they otherwise would have been. Thus, paired males can benefit from being cuckolded by relatives provided that cuckoldry is inevitable, or at least highly likely, as it is in our study system. The consequences of male-cuckolder relatedness have only recently begun to be appreciated [6, 42], and it remains to be investigated how common male-cuckolder relatedness is across taxa and whether it plays a general role in shaping mating systems.

## Methods

### Study species

*V. moorii* is a small herbivorous cichlid endemic to Lake Tanganyika in East Africa. Male-female pairs defend shallow rocky territories ranging in size from 1 to 4 m^2^, where they deposit eggs and raise broods of fry [26–28]. Parents raise one brood at a time for approximately 100 days [57], during which time they defend their young against territorial intrusions from con- and hetero-specifics [27]. Although the *V. moorii* mating system is characterized by extreme paternity loss, paired males engage in very little cuckoldry themselves as the vast majority of cuckoldry is perpetrated by unpaired males in the population [31].

### Field collections and microsatellite genotyping

We identified *V. moorii* territories within a study quadrat (~ 100 m × ~ 50 m; depth range, 1.7–12.1 m) in the south of Lake Tanganyika, Zambia (8° 42′ 29.4″ S, 31° 07′ 18.0″ E) over the course of two sampling excursions, from September 22 to October 28, 2015 (corresponding with the dry season), and from April 4 to 20, 2016 (corresponding with the rainy season). The genetic samples collected from these field seasons have been previously analysed by Bose et al. [31] to investigate patterns of extra-pair paternity between paired and unpaired males. In the current study, however, we use these data to answer a very different set of research questions. See ref. [31] for more detailed descriptions of sampling methods and genetic analyses.

In brief, we identified parental care-giving pairs of *V. moorii* in a study quadrat in the field while on scuba. The parents were captured using gill nets (though several individuals evaded capture), measured for total length (to the nearest 0.1 cm), fin clipped, and released back to their territories. Furthermore, for each adult pair, we measured the distances from their territory to their nearest 2–3 neighbors’ territories (center to center) and used this to generate a map showing the locations of, and relative distances between, territories. During the dry season, we sampled paired males from 74 territories and paired females from 85 territories. During the rainy season, we sampled paired males from 45 territories and paired females from 49 territories. We also captured fry from each care-giving pair and sacrificed them in MS-222 (1 g/L lake water). For this study, we included 821 fry from 32 pairs in the dry season, and 1129 fry from 38 pairs in the rainy season. Fry and fin clips were stored in 99.9% ethanol and transported back to the lab for parentage analyses using microsatellite genotyping. This work was carried out with the permission of the Fisheries Department of Zambia and under a study permit issued by the government of Zambia.

We extracted DNA from fry tissues using a standard Chelex protocol [58] and from fin clip tissue using an ammonium acetate precipitation protocol [59]. All adult fin clips and most fry were genotyped at 14 microsatellite loci though 4 broods (138 fry in total) were genotyped at 9 microsatellite loci only. The microsatellite loci used were Pmv17 [60], TmoM11 [61], Pzeb3 [62], UNH2075 [63], Ppun21 [64], Ppun9 [64], Hchi59 [65], Hchi94 [65], UME002 [66], Pmv13 [60], UME003 [66], UNH908 [67], Ppun5 [64], and Ppun20 [64]. These markers were highly polymorphic, with an average of 21.2 alleles per locus and a mean expected heterozygosity of 0.88. Exclusion probability (assuming the mother was known) calculated across loci amounted to 0.9999997 (calculated in GERUD2 [68]). All loci complied with Hardy–Weinberg expectations after correcting for multiple testing (see ref. [31] for additional details).

### Parentage analyses and construction of cuckolder genotypes

Parentage analyses were conducted with the aid of COLONY (v 2.0.6.1, ref. [69]), using population allele frequencies obtained from the adults captured in each season (*N* = 219 in the dry season, *N* = 98 in the rainy season). COLONY used the multi-locus genotypes of the care-giving adults as well as the sampled fry to identify fry as either within-pair or extra-pair, and then split the extra-pair fry into full-sib groups that were each sired by a different extra-pair male (see ref. [31] for details). With the maternal genotypes known, we could also construct the multi-locus genotypes of cuckolder males based on the extra-pair fry that they had sired. Generally, for each group of extra-pair full-sibs, the non-maternal alleles at each locus were assigned to the cuckolder father. However, when cuckolders sired too few offspring, their genotypes could not be fully reconstructed, and we focused only on cuckolders for which we could unambiguously identify both alleles for at least 10 of the 14 loci (although note that our relatedness estimates between parents and offspring take all cuckolders into account: see below). When only one paternal allele at a locus could be identified in a given a full-sib group, it was unclear whether the cuckolder father was homozygous at that locus or whether the fry inherited only one of his alleles. In this case, our assignment of alleles depended on how many extra-pair fry the cuckolder had sired; if the male had sired fewer than eight fry, we scored his genotype as being potentially heterozygous (i.e. ‘paternal allele observed in offspring’/‘unknown’), but if he had sired at least eight fry, we scored his genotype as being homozygous for the observed paternal allele. Furthermore, when the mother and an extra-pair fry shared the same heterozygous genotype at a locus, for example X/Y, it was not possible to identify which allele was inherited from the mother or the cuckolder father. In this case, the paternal allele had two possibilities, either X or Y, and examination of additional extra-pair fry genotypes was required to resolve this ambiguity. However, if the ambiguity persisted after assessing all of the cuckolder’s fry, we assigned the paternal allele as the one that had a higher frequency within the population (unless the difference in population allele frequencies was < 1%, in which case the allele was recorded as ‘unknown’). Thus, we attained multi-locus genotypes directly for paired individuals and indirectly for cuckolders, provided that the cuckolders fertilized a sufficient number of offspring. We then used the ‘Demerelate’ r package (v. 0.9 – 3, ref. [70]) to calculate two symmetrical estimates of relatedness between all individuals in our dataset: *r*_QG_ (following ref. [71]) and *r*_LR_ (following ref. [72]). We used both estimators, *r*_QG_ and *r*_LR_, because simulations based on the allele frequencies in our dataset showed that *r*_LR_ outperformed other indices for unrelated pairs, whereas *r*_QG_ did better for closely related pairs (as shown previously in refs [73, 74].).

### Do *V. moorii* exhibit more reproduction among relatives than expected under random mating?

To begin, we tested whether relatedness estimates differed between the two seasons (i.e. sampling excursions). We used two linear models to test whether relatedness within pairs (estimated by *r*_QG_ and *r*_LR_) differed between the dry and rainy seasons. We similarly used two linear mixed effects models (LMMs, ‘lme4’ r package, v. 1.1 – 16, ref. [75]) to test whether relatedness between paired females and their extra-pair (cuckolder) males differed between the seasons. We included ‘Female ID’ as a random intercept term to account for some females mating with multiple cuckolder males. Lastly, we also used two LMMs to test whether the relatedness between paired males and their cuckolders differed between the seasons. We included ‘Paired male ID’ as a random intercept to account for some males being cuckolded by multiple extra-pair males. We applied Yeo-Johnson power transformations where necessary to improve normality of the models’ residuals. Overall, we found no significant differences between the seasons (all *p* ≥ 0.37). We therefore pooled our samples from both seasons for the remainder of our analyses unless otherwise specified.

We used a series of randomization tests to test for elevated pairwise relatedness between all three parties in the *V. moorii* mating system: paired males, paired females, and cuckolder males. In particular, we were interested in whether the (i) mean, (ii) variance, and (iii) skewness of our observed distributions of pairwise relatedness estimates were greater than would be expected under random mating. For example, a distribution of observed relatedness estimates with an elevated mean would suggest that *V. moorii* pair with (or cuckold) relatives more often than expected by chance. On the other hand, an observed distribution with elevated variance and positive skewness would specifically indicate that there were more *high-relatedness* pairings in the observed data than would be expected by chance. In combination, all three measures (mean, variance, and skewness) give a comprehensive understanding of the pairwise relatedness structure between individuals in a population.

To test for elevated relatedness within social pairs, we first calculated *r*_QG_ and *r*_LR_ between the paired males and females that we observed in the field during the two seasons. We then recorded the mean, variance, and skewness of the observed distribution of relatedness estimates (‘moments’ r package, v. 0.14, ref. [76]). Next, we simulated random pairings between the males and females. We randomly assigned our observed males and females to new partners (i.e. re-sampling without replacement) creating the same number of social pairs as in our observed dataset though we ensured that random pairs were only formed between individuals sampled during the same season. Again, we calculated *r*_QG_ and *r*_LR_ from each new pairing and recorded the mean, variance, and skewness from the resulting distribution. We repeated this randomization process 10,000 times to create null distributions against which to compare our observations. We computed *p* values as the proportion of the randomized trials that yielded mean, variance, and skewness values *more* extreme than our observed data.

We followed a similar process to test for elevated relatedness between paired females and cuckolder males. We first calculated *r*_QG_ and *r*_LR_ between our observed paired females and each one of their extra-pair males (as some females spawned with multiple cuckolders). Note again that the cuckolders used here had each sired a sufficient number of offspring for us to reconstruct their genotypes. We then took the average of the relatedness estimates between each female and her *set* of extra-pair males (i.e. cuckolder males). From this data, we recorded mean, variance, and skewness. We then randomly assigned a new set of extra-pair males to each paired female in our observed data. Again, we ensured that individuals could only be assigned together if they were sampled during the same season. Furthermore, it is exceedingly rare for individual cuckolder males to successfully cuckold in more than one territory over the time span of a single brood cycle (approximately 100 days, ref. [31]), and so for each permutation of our data here, we assumed that each cuckolder male would sire offspring with only a single female. We also ensured that each reshuffling of the data preserved the distribution of extra-pair males per female that we originally observed in each season. We took the average of the relatedness estimates between each female and her new set of randomly assigned extra-pair males, and then, we recorded mean, variance, and skewness. This randomization process was repeated 10,000 times, and *p* values were computed as the proportion of randomized trials yielding mean, variance, and skewness values *more* extreme than our observed data. It is important to note that for this randomization test, we omitted broods where the paired male had 0% paternity (6 out of 70 broods). This was done because we could not be sure whether these incidents indicated 100% cuckoldry or a territory takeover. Omitting these broods ensured that, in the event of a takeover, we would not mistake the previous paired male for a cuckolder. We followed a similar procedure to test for elevated relatedness between paired males and their set of cuckolder males. We similarly omitted broods of 0% paternity in order to guarantee that our analyses compared relatedness between males and cuckolders that were both present at the time of spawning. Due to multiple comparisons, we used the Benjamini–Hochberg procedure for controlling false discovery rates following ref. [77], setting the false discovery rate to 10% [78].

### Does parent-offspring relatedness deviate from expectations?

We calculated *r*_QG_ and *r*_LR_ between paired adults and each of the within-pair and extra-pair fry under their care. If paired males were related to their female partners, then we would expect their relatedness estimates to within-pair offspring to exceed 0.5. To test this, we first subtracted 0.5 from the relatedness estimates between the paired males and each of their within-pair offspring. We then tested for a significant intercept effect using two intercept-only LMMs, one for *r*_QG_ and the other for *r*_LR_. We included ‘Brood ID’ as a random intercept in each model. Next, if paired females were related to their extra-pair mates, then we would expect female relatedness to their extra-pair offspring to exceed 0.5. To test this, we subtracted 0.5 from the relatedness estimates between the paired females and each of their extra-pair offspring. Again, we fit two intercept-only LMMs. Here, we included the identity of each offspring’s genetic sire as a random intercept nested within Brood ID. Finally, if paired males were related to their cuckolders, then we would expect their relatedness to extra-pair fry to exceed 0 *after* accounting for any deviation from random allele sharing occurring within their pair bond. Here, we subtracted ½*R* from each relatedness estimate between the paired males and the extra-pair offspring in their brood, where *R* is the relatedness estimate between the paired male and female. Note that R could be positive or negative depending on whether the social pair shared more or fewer alleles than expected by chance. We then fit two intercept-only LMMs, similarly including the identity of each offspring’s genetic sire as a random intercept nested within Brood ID. We omitted broods of 0% paternity for these analyses for the same reasons outlined above.

### Is there spatial structure in the relatedness between males in the field?

Bose et al. [31] recently showed that the vast majority of cuckoldry in the *V. moorii* system is perpetrated by unpaired males in the population. We therefore tested whether unpaired males in the population were caught in close proximity to any of their male relatives that were pair bonded. Our sampling regime during the dry season (October sampling excursion) also included the opportunistic capture of 40 unpaired males in addition to the paired males that we sampled. Unpaired males that were observed to be in close proximity to certain territories were captured opportunistically, fin-clipped, and genotyped. We used the distances that we measured between territories within the study quadrat and a field-drawn sketch of nest locations to calculate the relative locations of all nests using trigonometric principles. From these locations, we generated a matrix describing the pairwise distances between territories and between males (in Mathematica version 11.2, Wolfram Research, Inc.). For the purposes of this analysis, the spatial positions of our unpaired males were assumed to be the same as the territories that they were captured next to. Next, we generated another matrix to describe the pairwise relatedness estimates, *r*_QG_ and *r*_LR_, between each unpaired male and each paired male in the study quadrat. In order to test whether unpaired males remain in close proximity to paired male relatives, we conducted a series of permutation tests. First, we took the maximum relatedness estimate (*r*_QG_ and *r*_LR_) that we could find between each unpaired male and the paired males within an X m radius. We then subtracted the maximum relatedness estimate between each unpaired male and the paired males *beyond* the X m radius. We took the average of these Δ*r* values as our observed test statistic. We then permuted the spatial positions of unpaired males in our sample by randomly assigning each unpaired male to a territory within the quadrat. In each permutation round, territories were able to receive up to one unpaired male, if we had originally sampled no unpaired males next to them, or up to the number of unpaired males that we had originally captured by them. We then calculated Δ*r* at radius *X* m around each unpaired male as described above and repeated this process 10,000 times. We computed *p* values as the proportion of randomized trials yielding Δ*r* values more extreme than our observed data. We performed this test starting at *X* = 1 m and then repeated it every 1 m increment up to a radius of 10 m. Note that for two territories, the paired male was replaced by a new paired male during our sampling period. Because we could not know the exact date when this switch occurred in relation to when we captured most of the unpaired males in the quadrat, we opted for a conservative approach and omitted these two paired males as well as the four unpaired males that were captured alongside them from these analyses. Thus, these analyses were conducted on 36 unpaired males and 72 breeding territories/paired males that we had genotyped. This omission does not qualitatively change our results. Because of the multiple comparisons here, we used the Benjamini–Hochberg procedure and set the false discovery rate to 10% [77].

Lastly, we generated relatedness matrices describing the pairwise relatedness estimates, *r*_QG_ and *r*_LR_, between all paired males in the study quadrat during the dry season. Using these matrices as well as our distance matrix, we tested for any spatial structure in relatedness between the paired males in our study quadrat (i.e. increasing or decreasing relatedness with spatial separation between territories). For this, we used Mantel tests (vegan r package, ref. [79]), each based on 10,000 random permutations of the data, and we performed separate Mantel tests for *r*_QG_ and *r*_LR_ estimates.

## Additional file

Additional file 1: Supplementary Materials, **Figures S1-S2, Table S1**. Details on an extension of the mathematical model in which we relax a prior assumption, and details on our calculation of paired male fitness increase due to their relatedness to cuckolders. **Figure S1.** Null distributions of variance and skewness for r_QG_ and r_LR_. **Figure S2.** Lack of a relationship between spatial separation and genetic relatedness between paired males in our study quadrat. **Table S1.** showing the results of our randomization tests on the mean, variance, and skewness of r_GQ_ and r_LR_ estimates for 1) paired male vs. cuckolders, 2) paired female vs. cuckolders, and 3) paired male vs. paired female relationships. (DOCX 477 kb)
