# Supplementary material for: Inclusive fitness benefits mitigate costs of cuckoldry to socially paired males
Source: BMC Biol. 2019 Jan 31;17:2. doi: 10.1186/s12915-018-0620-6 (PMC6354359; doi:10.1186/s12915-018-0620-6)
Supplement: Supplementary file 1 — Supplementary Materials, Figures S1-S2, Table S1. Details on an extension of the mathematical model in which we relax a prior assumption, and details on our calculation of paired male fitness increase due to their relatedness to cuckolders. Figure S1. Null distributions of variance and skewness for rQG and rLR. Figure S2. Lack of a relationship between spatial separation and genetic relatedness between paired males in our study quadrat. Table S1. showing the results of our randomization tests on the mean, variance, and skewness of rGQ and rLR estimates for 1) paired male vs. cuckolders, 2) paired female vs. cuckolders, and 3) paired male vs. paired female relationships. (DOCX 477 kb) [file 12915_2018_620_MOESM1_ESM.docx]

SUPPLEMENTARY MATERIALS


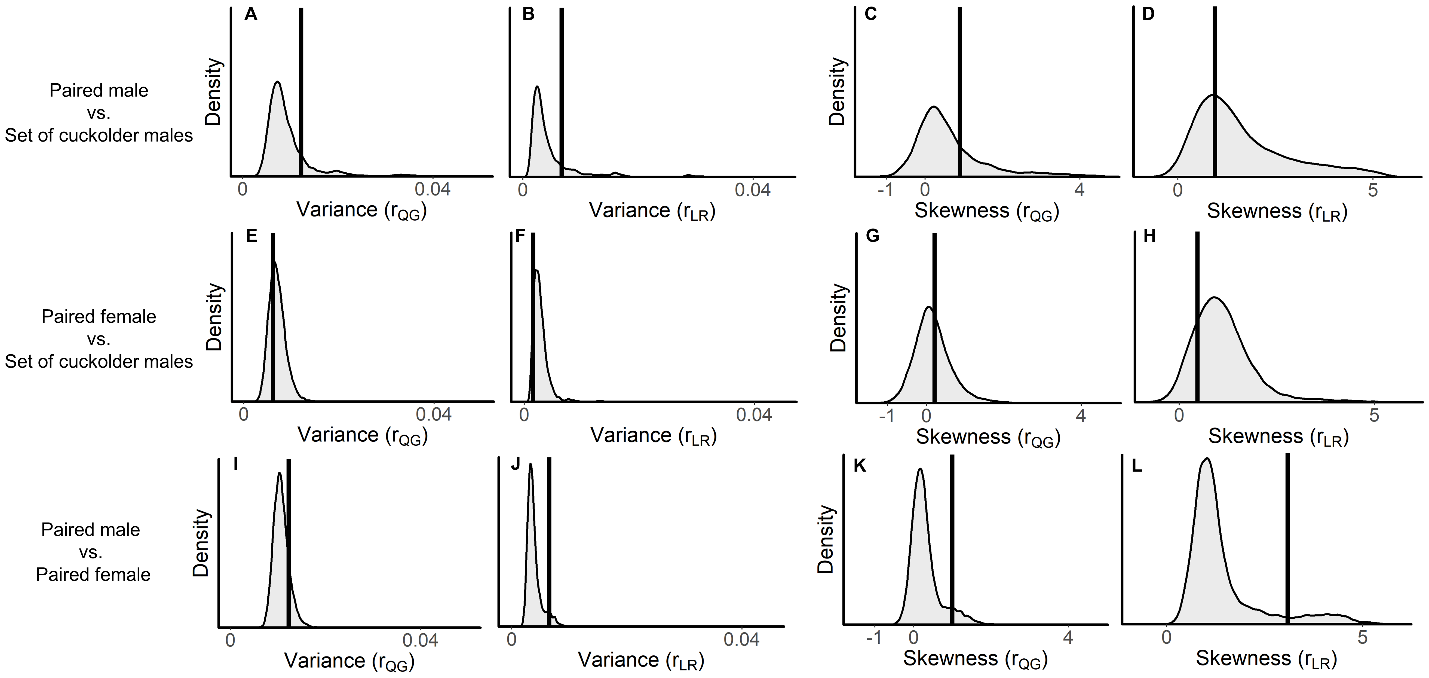


**Figure S1:** Density plots showing null distributions for the variance and skewness values of the pairwise relatedness estimates derived from the randomization tests described in the methods. Vertical black bars indicate our observed values.


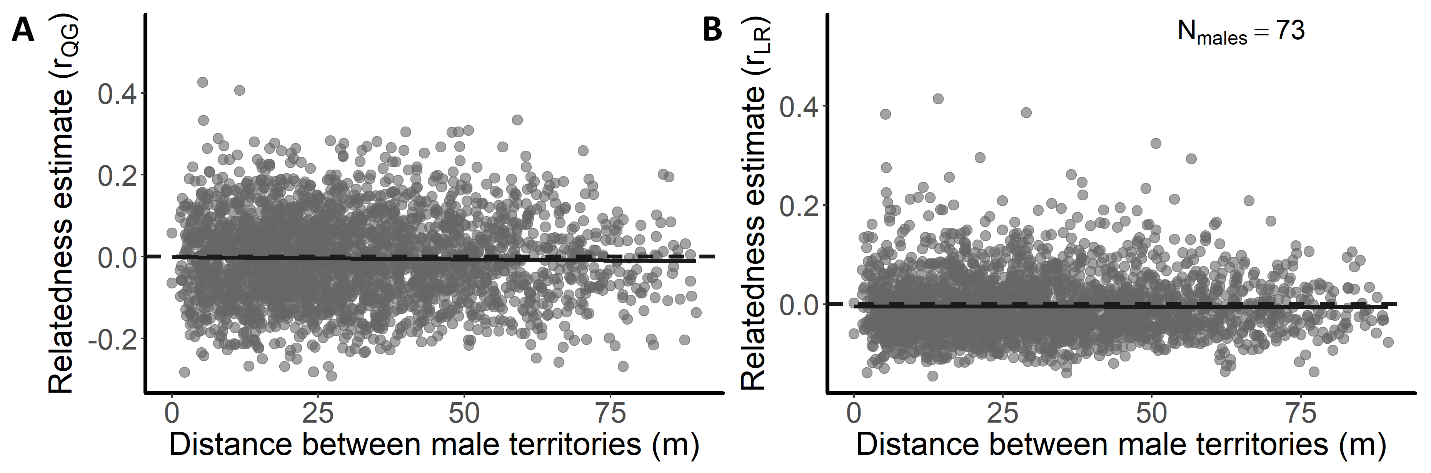


**Figure S2:** Pairwise relatedness estimates, r_QG_ (A) and r_LR_ (B), between paired, territory-holding *V. moorii* males sampled during the dry season (October 2015). Horizontal dashed lines indicate relatedness = 0, while solid lines show linear regression fits.

**Table S1:** Summary of permutation tests of Δr, where Δr is the maximum relatedness between unpaired males and the paired males within an X m radius of them *minus* the maximum relatedness between the unpaired males and the paired males at or beyond the X m radius (but still within the study quadrat). Radii of X = 1, 2, 3, …10 m were tested. Data were collected from our study quadrat in October 2015. *P*-values marked with an * indicate statistically significant results after applying the Benjamini-Hochberg Procedure for controlling false discovery rates, here set to 10%. Note that there were two unpaired males for which we did not have a genotyped paired male within 2 and 3 m respectively.

| **Distance radius (m)** | **Average (± SD) no. of**  **paired males *within* radius of unpaired males** | **Average (± SD) no. of**  **paired males at or *beyond* radius of unpaired males** | **Δr_QG_, *p*** | **Δr_LR_, *p*** |
| --- | --- | --- | --- | --- |
| 1 | 0.9 ± 0.2 | 68.1 ± 0.2 | -0.29,  *p* = 0.95 | -0.25,  *p* = 0.98 |
| 2 | 1.3 ± 0.7 | 67.7 ± 0.7 | -0.24,  *p* = 0.45 | -0.21,  *p* = 0.28 |
| 3 | 2.2 ± 1.3 | 66.8 ± 1.3 | -0.21,  *p* = 0.38 | -0.19,  *p* = 0.24 |
| 4 | 2.9 ± 1.4 | 66.1 ± 1.4 | -0.18,  *p* = 0.26 | -0.16,  *p* = 0.068 |
| 5 | 3.7 ± 1.8 | 65.3 ± 1.8 | -0.18,  *p* = 0.64 | -0.14,  *p* = 0.091 |
| 6 | 5.2 ± 2.1 | 63.8 ± 2.1 | -0.11,  *p* = 0.027* | -0.091,  *p* = 0.0031* |
| 7 | 6.0 ± 2.4 | 63.0 ± 2.4 | -0.11,  *p* = 0.063 | -0.087,  *p* = 0.0076* |
| 8 | 7.1 ± 2.9 | 61.9 ± 2.9 | -0.082,  *p* = 0.010* | -0.074,  *p* = 0.0052* |
| 9 | 7.8 ± 3.2 | 61.2 ± 3.2 | -0.069,  *p* = 0.0045* | -0.063,  *p* = 0.0038* |
| 10 | 8.8 ± 3.8 | 60.2 ± 3.8 | -0.067,  *p* = 0.0091* | -0.058,  *p* = 0.0050* |

DETAILS OF THE MATHEMATICAL MODEL

*Multiple related cuckolders*

Here we consider the case where a paired male has multiple male relatives who may potentially cuckold him. For simplicity, however, we retain the assumption that at most one such male targets any particular spawning event (i.e. the identity of the related cuckolder may change from one spawning event to another). This assumption will hold approximately if each paired male has few related cuckolders, or the probability of any one related cuckolder attending a spawning event is low, or both. We write $\bar{r}_{PC}$ for the average relatedness coefficient between the paired male and his set of related cuckolders. As in the main text, the average relatedness between paired males and unrelated cuckolders is assumed to be zero.

The direct fitness of the paired male (equations 2 and 3 in the main text) is unchanged in this scenario. To calculate his indirect fitness, we can simply replace $r$ with $\bar{r}_{PC}$ in equations 4 and 5. The probability $f$ of a related cuckolder being present at a given spawning may also be higher when there are multiple such cuckolders present in the population. The results of Figure 4A still apply if $r$ is replaced with $\bar{r}_{PC}$ (keeping in mind that this panel is drawn with $f=0.5$).

We assume that paired males pay a uniform cost to discriminate relatives from non-relatives, regardless of whether a relative is actually present. In contrast, cuckolders may be able to decide which males to cuckold on a case-by-case basis. Under this assumption, the results in Figure 4B are unchanged if we interpret $r$ as the relatedness coefficient $r_{CP}$ between the focal cuckolder and any paired male that he might target. If there is at most one related cuckolder, as in the main text, then we have $r=\bar{r}_{PC}=r_{CP}$ (note that Figure 4C assumes this condition).

*Average relatedness of paired male to his cuckolders*

Here we derive the average relatedness of a paired male to all of his successful cuckolders (both related and unrelated). For a paired male that defends indiscriminately, the probability that a least one cuckolder is successful in gaining paternity is given by:

| $q^{i}=\left( 1-f \right)\sum_{k=1}^{\infty} s_{k}\left( \mu_{C},\mu_{D} \right)+f\sum_{k=0}^{\infty} s_{k}\left( \mu_{C},\mu_{D} \right)$ | [S1] |
| --- | --- |

For a discriminately defending male, the probability is similarly:

| $q^{d}=\left( 1-f \right)\sum_{k=1}^{\infty} s_{k}\left( \mu_{C},{\left( 1-a \right)\mu}_{D} \right)+f\sum_{k=0}^{\infty} s_{k}\left( \mu_{C},{\left( 1-a \right)\mu}_{D} \right)$ | [S2] |
| --- | --- |

The mean relatedness of an indiscriminate paired male to his cuckolders is consequently:

| $r^{i}=\frac{{f\cdot\bar{r}}_{PC}}{q^{i}}\sum_{c=0}^{\infty} p_{c}\left( \mu_{C} \right)\sum_{d=0}^{c} p_{d}\left( \mu_{D} \right)\frac{1}{c+1}$ | [S3] |
| --- | --- |

For a paired male that defends discriminately, the average relatedness to his cuckolders is similarly:

| $r^{d}=\frac{{f\cdot\bar{r}}_{PC}}{q^{d}}\sum_{c=0}^{\infty} p_{c}\left( \mu_{C} \right)\sum_{d=0}^{c} p_{d}\left( (1-a)\mu_{D} \right)\frac{1}{c+1-d}$ | [S4] |
| --- | --- |

CALCULATION OF AVERAGE PAIRED MALE FITNESS INCREASE DERIVED FROM RELATEDNESS WITH CUCKOLDERS

On average, paired males gain 55.1% paternity per own-brood that they sire offspring within (ref. ^31^). This means that 44.9% paternity per brood is lost to cuckolders. Paired males are also related to their cuckolders at a level of r_QG_ = 0.038.

Thus, accounting for male-cuckolder relatedness, the adjusted paternity estimate for an average paired male to his brood is:

| $adjusted paternity=0.551+0.449(0.038)=0.568$ |  |
| --- | --- |

This adjusted paternity estimate is 3.1% higher than the paternity estimate calculated by ignoring male-cuckolder relatedness:

$$\frac{0.568}{0.551}=1.031$$
